# A Rare Case of Adult Acquired Flatfoot Deformity (AAFD) With Chronic Atraumatic Subtalar Dislocation: Successful Reduction and Fusion of the Talonavicular and Talocalcaneal Joints

**DOI:** 10.7759/cureus.97680

**Published:** 2025-11-24

**Authors:** Ilias Fanourgiakis, Emmanouil Zervos, Anastasios Mourikis, Alkiviadis Vossos

**Affiliations:** 1 3rd Orthopedic Department, KAT General Hospital, Athens, GRC; 2 Orthopedic Department, Athens Medical Center, Athens, GRC

**Keywords:** adult-acquired flatfoot deformity, posterior tibial tendon dysfunction, subtalar dislocation, talocalcaneal, talonavicular joint

## Abstract

Chronic atraumatic subtalar dislocation is an exceptionally rare and severe manifestation of adult acquired flatfoot deformity (AAFD) secondary to posterior tibial tendon insufficiency (PTTI). Early recognition is critical, as progressive deformity leads to rigid valgus malalignment and functional decline. A 65-year-old male presented with a seven-year history of worsening left foot pain and deformity. Examination showed rigid hindfoot valgus, midfoot collapse, forefoot abduction, and taut skin over the medial talar head. Radiographs revealed chronic lateral subtalar dislocation with talonavicular and talocalcaneal involvement. Surgical reduction and fusion were performed, utilizing three cannulated cancellous screws for the talocalcaneal joint and two bone staples for the talonavicular joint, followed by structured rehabilitation. At six months, the patient demonstrated substantial improvement in pain, function, and radiographic alignment, with stable fusion. This case highlights the importance of recognizing severe AAFD with subtalar dislocation and demonstrates that joint reduction with selective fusion can yield significant functional improvement.

## Introduction

Subtalar dislocation is a rare condition, usually following high-energy trauma and accounting for less than 1% of all joint dislocations. Lateral subtalar dislocation, resembling "acquired flat foot" post-injury, occurs in 15%-35% of cases, highlighting the clinical challenge it presents [1-4]. Atraumatic cases are exceedingly uncommon and typically reflect severe progression of adult acquired flatfoot deformity (AAFD) secondary to posterior tibial tendon insufficiency (PTTI). As medial soft tissues attenuate, the calcaneus shifts laterally and into valgus, producing rigid deformity, peritalar subluxation (PTS), and in exceptional cases, chronic subtalar dislocation [5,6]. Chronic atraumatic subtalar dislocation represents the extreme end of the AAFD spectrum and requires careful recognition, as prolonged deformity results in joint erosion, ligament failure, and fixed malalignment [7]. We present a rare case of chronic atraumatic subtalar dislocation secondary to PTTI managed with successful reduction and fusion, contributing valuable insight into surgical decision-making for severe hindfoot deformities [8].

## Case presentation

Informed consent was obtained for the publication of this case report. A 65-year-old male patient presented with a seven-year history of progressive, non-traumatic left foot pain and progressive deformity (midfoot collapse and forefoot abduction). Despite custom orthotics and bracing, his symptoms persisted.

**Figure 1:**
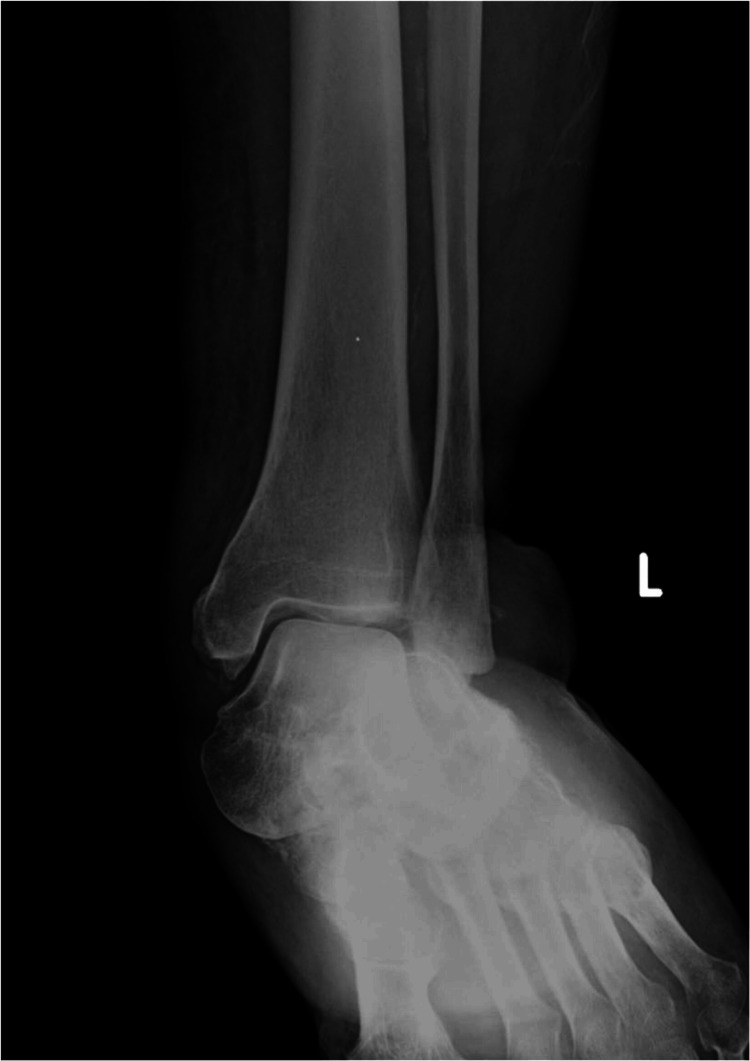
Preoperative anteroposterior ankle X-ray

**Figure 2:**
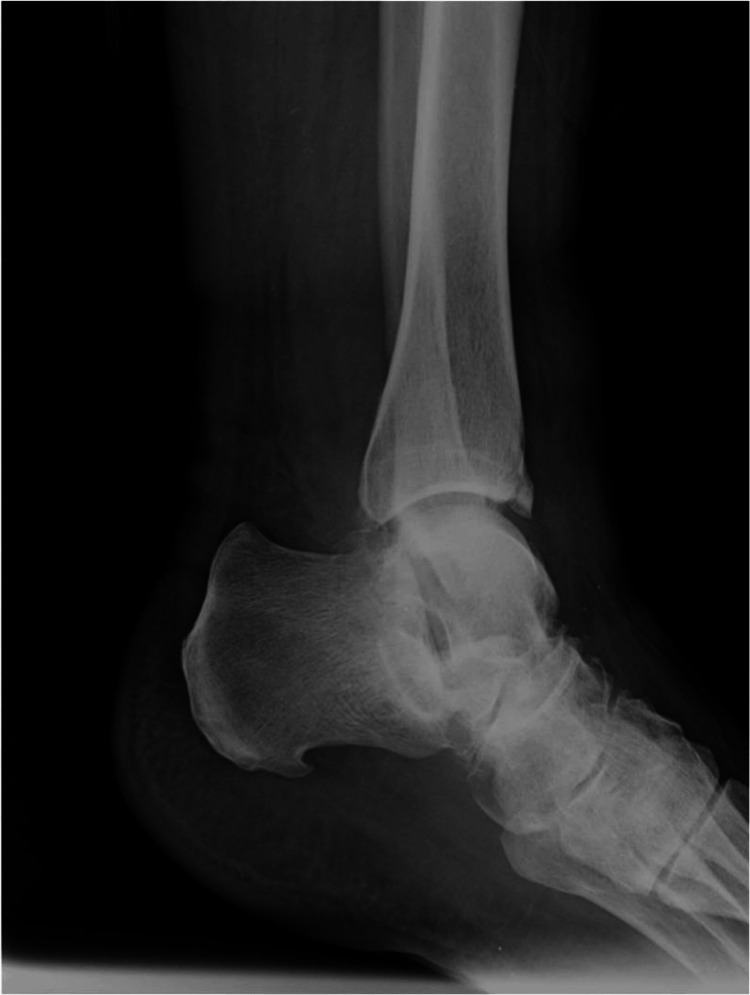
Preoperative lateral ankle X-ray

Clinical examination upon presentation revealed tenderness at the talonavicular joint and tautness of the skin over the medial aspect of the talar head. Diagnostic imaging confirmed lateral displacement of the calcaneus, indicative of lateral subtalar dislocation involving the talonavicular and talocalcaneal joints, secondary to PTTI (Figures 1, 2).

The preoperative Foot Function Index (FFI) score was 82 (indicating severe disability), and the visual analog scale (VAS) for pain was rated at 8 (indicating high pain intensity). These assessments underscored the need for surgical intervention to address the patient's complex foot deformity and associated symptoms.

**Figure 3:**
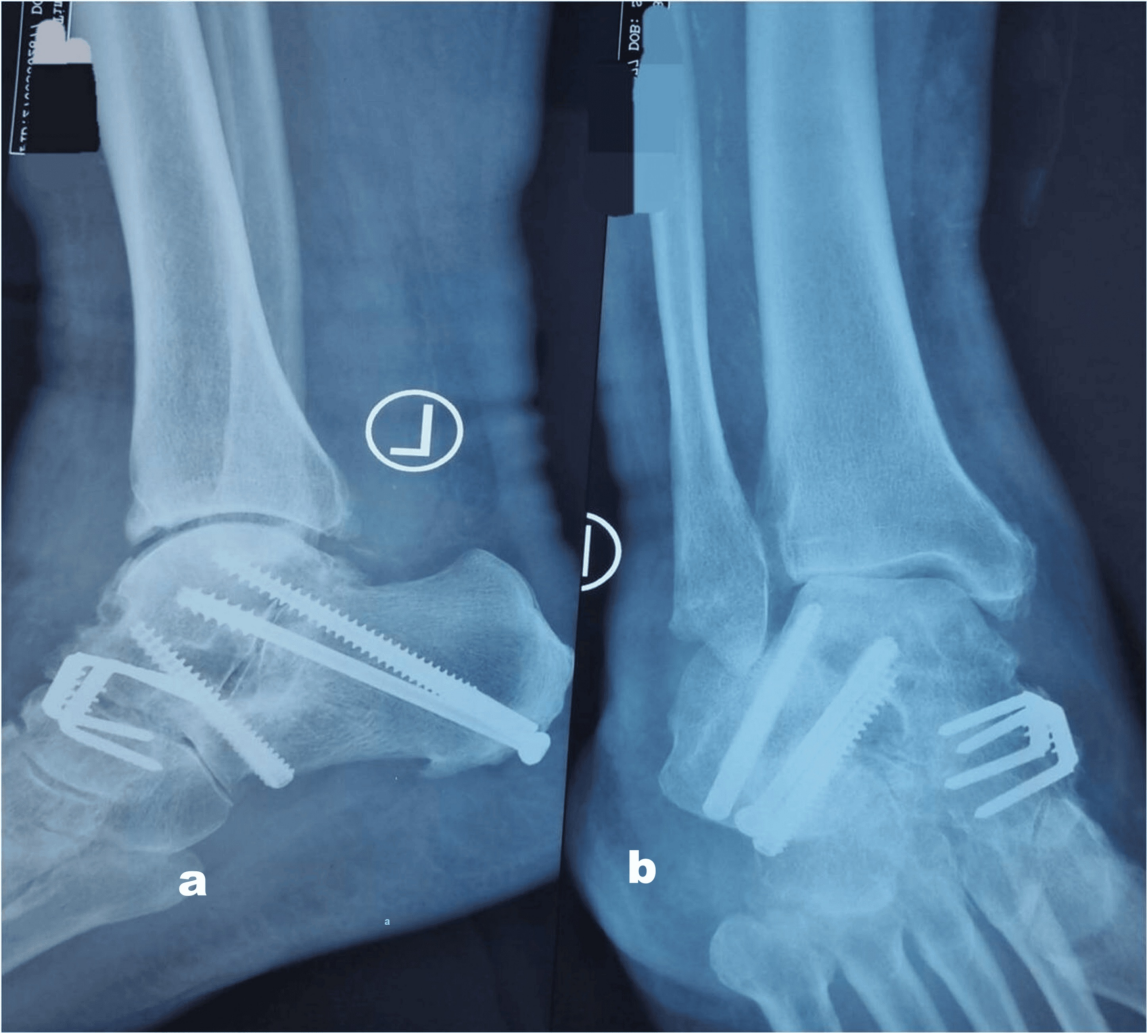
a) Postoperative lateral ankle X-ray. b) Postoperative anteroposterior ankle X-ray

The surgical strategy involved a two-incision approach to optimally address the deformity and facilitate joint fusion. The first incision was made laterally to access the talocalcaneal joint, followed by a medial incision for talonavicular joint preparation. A distractor facilitated full exposure of the talonavicular joint. Intraoperatively, residual cartilage was excised, and the subchondral bone was drilled, preserving the bone architecture. The talus was repositioned and reduced. The talocalcaneal joint was stabilized using three cannulated cancellous screws, while the talonavicular joint was fixed with two bone staples, ensuring robust correction of the deformity (Figures 3a, 3b).

Postoperatively, the patient's foot and ankle were immobilized in a splint for eight weeks to support healing. After removal of the splint, the patient embarked on a mobilization protocol involving progressive passive and active range of motion (ROM) exercises, subtalar-neutral gait training, and strengthening of the peroneals and intrinsic muscles. At 10 weeks postoperatively, the patient was advised to begin full weight-bearing with ankle support.

Three months following surgery, radiographic evaluation confirmed bony fusion of the talocalcaneal and talonavicular joints. The lateral subtalar dislocation showed marked improvement under weight-bearing conditions, though some degree of flatfoot deformity persisted. The adduction deformity exhibited notable improvement in frontal view assessments.

**Figure 4:**
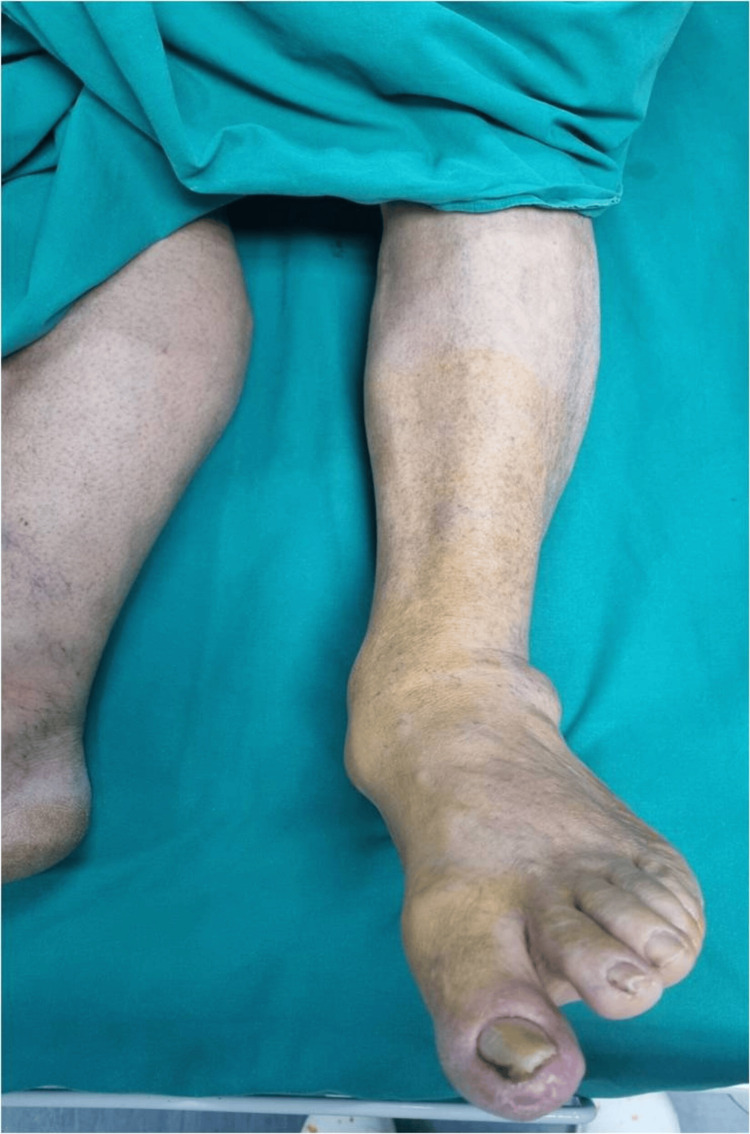
Clinical photograph illustrating the patient's foot before the surgical intervention

**Figure 5:**
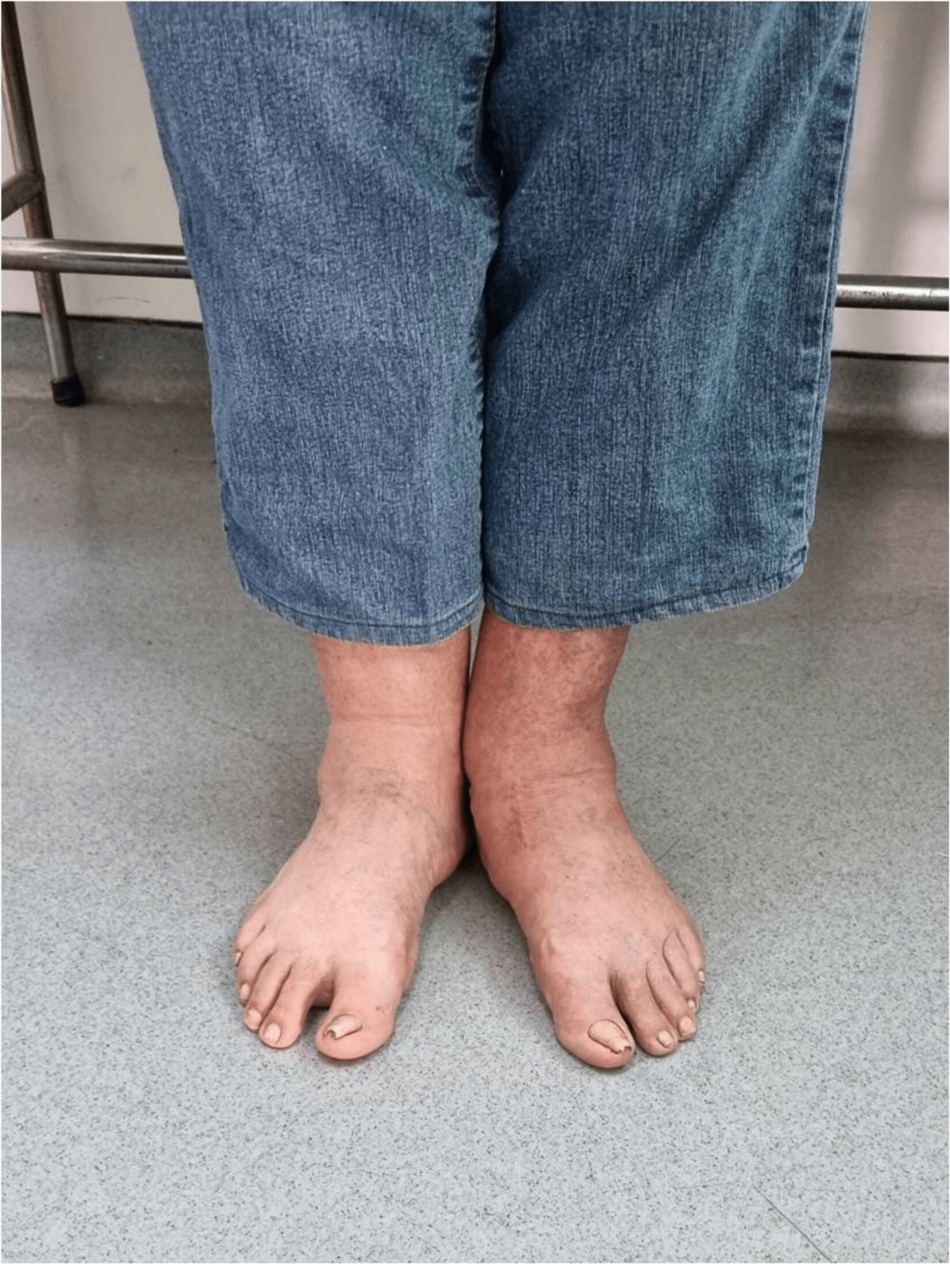
Clinical photographs of the patient's foot six months after surgical intervention, showing significant improvement in alignment and overall structure, correlating with the patient’s reported pain relief and improved function

At the six-month follow-up, the patient's FFI score improved to 30 (indicating substantial functional recovery), and the VAS for pain decreased to 2, reflecting significant pain relief (Figures 4, 5). These outcomes highlight the successful management of a complex foot deformity through a tailored surgical approach, leading to improved foot function, mobility, and patient quality of life.

Table 1 summarizes the timeline of clinical events.

**Table 1:** Timeline of clinical events FFI: Foot Function Index; PTTI: posterior tibial tendon insufficiency; ROM: range of motion; VAS: visual analog scale

Timepoint	Event
Pre-op	Chronic lateral subtalar dislocation involving the talonavicular and talocalcaneal joints, secondary to PTTI. Severe pain and deformity. VAS 8, FFI 82
Day 0	Surgical reduction and fusion of talonavicular+talocalcaneal joints
0-8 weeks	Splint immobilization
8-10 weeks	Progressive ROM, early weight-bearing with support
3 months	Radiographic union confirmed
6 months	VAS 2, FFI 30, improved alignment, and mild residual flatfoot

## Discussion

This case report presents a rare instance of chronic atraumatic subtalar dislocation secondary to PTTI in a 65-year-old male, managed successfully through reduction and fusion of the talonavicular and talocalcaneal joints. Unlike the more commonly discussed PTS in the context of AAFD, our case emphasizes the subtalar dislocation, underscoring the diversity within AAFD presentations and the potential for significant clinical challenge.

Chronic subtalar dislocations, particularly those without an acute traumatic history, pose unique diagnostic and treatment challenges. Unlike acute cases, where skin tension and risk of blistering or necrosis due to bony pressure are significant concerns, chronic cases like ours may present with more insidious symptoms and physical findings, such as tenderness over the talonavicular joint and taut skin over the medial talar head, without the acute skin complications [9-12].

The literature suggests that certain anatomical predispositions, such as the bony morphology of the talus and valgus angulation of the subtalar facet joint, may contribute to the development of AAFD [13,14]. Our patient's long-standing PTTI likely led to a gradual weakening and eventual disruption of the primary intrinsic ligaments of the subtalar joint, including the talocalcaneal interosseous and cervical ligaments, culminating in the observed dislocation [15]. This pathophysiology underscores the critical role of these ligaments in subtalar stability and highlights the necessity of addressing both tendon insufficiency and ligamentous support in treatment.

Surgical management in our case was guided by principles similar to those applied in traumatic lateral subtalar dislocations, focusing on restoring hindfoot alignment and correcting talonavicular uncoverage through joint fusion. The use of cannulated cancellous screws and bone staples provided stable fixation, facilitating early mobilization and weight-bearing, essential components of postoperative rehabilitation aimed at optimizing functional recovery.

Outcomes align with reports showing high satisfaction and union rates following subtalar or triple arthrodesis in rigid deformities. Residual flatfoot deformity, as in this case, is common and may require additional procedures in some patients. Outcome of subtalar arthrodesis, as reported by Pell et al., with high patient satisfaction and union rates, reflects the potential for positive long-term outcomes in cases like ours [16]. However, as noted, the persistence of flatfoot deformity postoperatively presents a limitation and an area for future consideration, possibly necessitating additional procedures such as calcaneal osteotomy or even triple arthrodesis for complete correction [16].

The absence of deltoid ligament reconstruction in our case aligns with some literature suggesting its potential benefit in improving outcomes in flatfoot deformity correction [17-19]. Future research may explore integrating deltoid ligament reconstruction into the surgical strategy for cases similar to ours, potentially enhancing postoperative alignment and functional outcomes.

The present study has certain limitations. It has a relatively short-term follow-up (six months), and the patient has mild residual pes planovalgus. Longer-term outcomes, including adjacent joint degeneration, remain unknown.

## Conclusions

This case highlights the successful surgical management of a rare chronic atraumatic subtalar dislocation secondary to PTTI. Reduction and fusion of the talonavicular and talocalcaneal joints restored alignment and significantly improved pain and function. While longer-term monitoring is necessary, this report provides valuable insight for clinicians managing severe AAFD with fixed subtalar deformity. Our findings contribute to the sparse literature on this condition and reinforce the importance of tailored surgical strategies in enhancing patient outcomes for complex foot deformities.
